# Factors associated with duration of breastfeeding in Bangladesh: evidence from Bangladesh demographic and health survey 2014

**DOI:** 10.1186/s12889-021-11804-7

**Published:** 2021-09-26

**Authors:** Ummay Ayesha, A. S. M. A. Mamun, Md. Abu Sayem, Md. Golam Hossain

**Affiliations:** 1 Health Research Group, Department of Statistics, University of Rajshahi, Rajshahi, 6205 Bangladesh; 2 Institute of Biological Sciences, University of Rajshahi, Rajshahi, 6205 Bangladesh

**Keywords:** Duration of breastfeeding, BDHS-2014, T-test, ANOVA, Multiple linear regression, Bangladesh

## Abstract

**Background:**

Breastfeeding for optimum duration is one of the most effective ways to reduce infant morbidity and mortality and confirms expected growth and development of children. The aim of this study was to determine the effect of socio-demographic and anthropometric determinants on duration of breastfeeding (DB) among mothers in Bangladesh.

**Methods:**

The data was extracted from the Bangladesh Demographic and Health Survey (BDHS)-2014. A total of 3541 married non–pregnant and currently non-breastfeeding Bangladeshi mothers in reproductive age who had at least one child aged 6–36 months were included in this study. Independent sample t-test and one-way analysis of variance (ANOVA) were used to find the significance difference in DB between two and more than two groups respectively. Multiple linear regression model was utilized to determine the effect of socio-economic, demographic, anthropometric and health related variables on DB.

**Results:**

This study revealed that the mean and median of DB among Bangladeshi mothers were 18.91 (95% CI: 18.65–19.17) and 19.00 months respectively. Independent sample t-test and ANOVA showed that DB among Bangladeshi mothers was significantly influenced by (i) ANC visits, (ii) religion, (iii) mode of delivery, (iv) place of delivery, (v) parents’ education, (vi) geographical location, (vii) mothers’ occupation and (viii) household wealth quintile. Multiple regression analysis demonstrated that mothers’ age, total number of children, mothers’ age at first birth, ANC visits, mothers’ occupation and geographical location were important predictors of DB.

**Conclusions:**

Healthcare providers and decision makers can consider these findings to make plan for counseling of mothers and family members to promote optimum DB practice in first 2 years of baby’s life.

## Background

Over the last decade, scientific studies have substantiated the evidence of the integral role of breastfeeding in the survival, growth and development of children, as well as good health and wellbeing of mothers. The World Health Organization (WHO) and the United Nations Children’s Fund (UNICEF) recommended that optimum early breastfeeding particularly within one hour after birth should be encouraged by healthcare professionals [1]. According to WHO, only breast milk can ensure a complete nutritional requirements for growth and development of babies in first six months [2]. Infants should be exclusively breastfed to achieve optimum growth, development and maintenance of health [2]. Furthermore, it is safe and contains antibodies that help to protect infants and boost immunity. Consequently, optimum breastfeeding reduces the risk of diarrhea, respiratory or ear infections and other infectious diseases that increase infant mortality [3]. Furthermore, optimal breastfeeding is also identified as a protective factor for overweight and obesity in childhood [4]. A study clearly mentioned that sub-optimal breastfeeding can increase the risk of mortality in first two or more years of child life [5]. In addition, breastfeeding is inexpensive, easily available, and clean at the right temperature. Breastfeeding also acts as natural family planning method and reduces the risk of developing breast and ovarian cancers [6]. Many of the health benefits of human milk are closely related, that is, the longer the baby receives human milk, the greater are the benefits. For adequate growth and maintenance of health, infants should also receive nutritionally rich and safe complementary foods along with breastfeeding from six months to two years of age [7, 8]. However, knowledge and attitude towards duration of breastfeeding among mothers are influenced by sociocultural, demographic and physiological factors such as education, income, residence, tradition, belief, and parents age [9–15].

This study was designed to work with the health related issues under the Sustainable Development Goals (SDGs). Breastfeeding practices for recommended duration are still sub- optimal in Bangladesh which would be a challenge to meet the SDGs by 2030*.* Subsequently*,* the benefits of breastfeeding would optimum when it continues for at least two years with complementary feeding [16]. To the best of our knowledge, there are a few studies on duration of breastfeeding practices in the context of Bangladesh by using BDHS-1999-2000 [17] and BDHS-2004 dataset [18]. None of these studies considered mothers’ BMI as a factor associated with duration of breastfeeding although some studies showed that BMI was significantly associated with breastfeeding status [19, 20]. Household wealth quintile, women education level and medical facilities have been increasing in Bangladesh during the last decades [21], which may have effect on knowledge, attitude and practice on duration of breastfeeding [22]. Therefore, it is important to investigate the duration of breastfeeding among mothers in Bangladesh considering the latest nationally representative data. The aim of the current study was to determine the effect of socio-economic, demographic and anthropometric variables on the duration of breastfeeding among mothers in Bangladesh.

### The study was based on the following hypotheses

H_01_: Socio-economic factors are significantly associated with duration of breastfeeding.

H_02_: Demographic factors have effect on the duration of breastfeeding.

H_03_: Duration of breastfeeding is associated with anthropometric measurements.

## Methods

### Study design and population

Bangladesh Demographic and Health Survey (BDHS)-2014 collected socio-demographic, health, anthropometric and lifestyle information from 17,863 Bangladeshi married women aged 15 to 49 years. The data was collected from March 24, 2014 to August 11, 2014. BDHS-2014 had taken information on duration of breastfeeding (DB) among their children born in the three years preceding the survey. This was a nationally representative survey which covered all administrative regions (divisions) of Bangladesh including both urban and rural settings. All information regarding study design, study population, data collection technique, instruments, data reliability, questionnaire etc. have been described elsewhere [22]. In our present study, we used BDHS-2014 data.

### Sampling

**Fig. 1:**
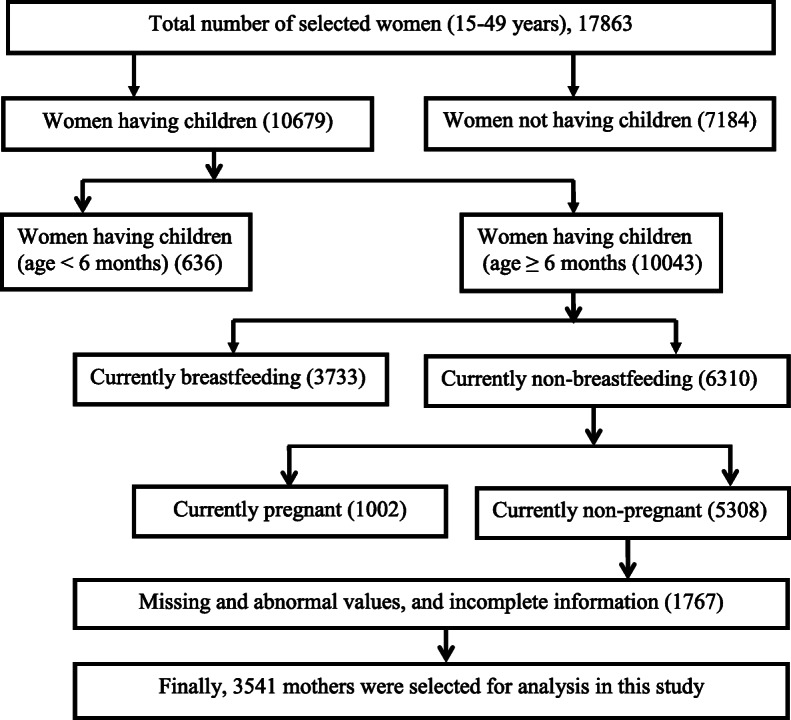
Sample selection procedure for the present study

In developing countries, the Demographic and Health Surveys (DHS) program is the main source for collecting and disseminating accurate, nationally representative data on health and population [23]. BDHS-2014 used two stage stratified random sampling for selecting sample from urban and rural areas from each administrative division. Bangladesh Bureau of Statistics (BBS) divided Bangladesh into many small areas called enumeration areas (EA) for population and housing census in 2011. BDHS-2014 considered EA as the primary sampling unit (PSU) for their survey. In the first stage, BDHS-2014 randomly selected 600 EAs (207 in urban and 393 in rural areas). In the second stage, they selected on average 30 households from each selected EA using systematic sampling. BDHS-2014 interview was successfully completed in 17,300 (99%) households. A total of 18,245 ever-married women in reproductive age were identified in these households and 17,863 were interviewed. From the preliminary sample, the mothers were excluded for the present study who had no children. The mothers who had children aged less than 6 and older than 36 months, and currently breastfeeding were also excluded from the present study. BDHS-2014 measured height and weight for all selected women, to avoid the bias of these measurements; we excluded all currently pregnant mothers from this study. Besides, abnormal values and incomplete information were also excluded from the data. Finally, 3541 mothers with their last born children were considered for this study (Fig. 1). Furthermore, BDHS-2014 collected information of one child if a mother had twin children [21].

### Inclusion criterion

Mothers’ having at least one child aged 6–36 months living with them, and currently not breastfeeding and non-pregnant were included in this study.

### Dependent variable

The DB among Bangladeshi mothers who had at least one child aged 6–36 months was the dependent variable for this study. To measure the variable, BDHS-2014 asked a question to every mothers, “how many months did you breastfeed of your last born children?”

### Independent variables: demographic and health related variable

Mothers’ age, antenatal care (ANC) visits during pregnancy), total children ever born, mothers’ age at first birth, sex of children, mode of delivery and place of delivery. Socio-economic variable: mothers’ education level, fathers’ education level, mothers’ occupation, geographic location (division), religion, place of residence, and household wealth quintile ((wealth index (WI)). Anthropometric variable: mothers’ body mass index (BMI). The categorical and continuous variables were given in Tables 1 and 2.

**Table 1:** Duration of breastfeeding among mothers by socio-demographic factors

Variable	Group	N (%)	Mean (in months)	SD	Value of t- statistic/F- statistic	***p***-value
**Demographic and health related variable**						
Antenatal care visits	Yes	2768 (78.2)	18.63	7.99	3.89	0.001
	No	773 (21.8)	19.89	8.00		
Sex of child	Male	1807 (51)	18.79	8.06	−0.90	0.366
	Female	1734 (49)	19.03	7.89		
Mode of delivery	Caesarean	831 (23.5)	18.27	7.98	2.64	0.008
	Vaginal	2710 (76.5)	19.10	7.97		
Place of delivery	Home	2163 (61.08)	19.18	7.98	2.54	0.011
	Hospital/Clinic	1378 (38.92)	18.48			
**Socio-economic variable**						
Mothers’ educational level	Uneducated	471 (13.3)	19.80	7.96	3.61	0.013
	Primary	975 (27.5)	19.15	8.13		
	Secondary	1705 (48.20)	18.66	7.90		
	Higher	390 (11.00)	18.27	7.85		
Fathers’ educational level	Uneducated	828 (23.4)	19.99	8.02	7.10	0.001
	Primary	1059 (29.9)	18.78	7.95		
	Secondary	1109 (31.3)	18.40	7.89		
	Higher	545 (15.4)	18.52	7.98		
Mothers’ occupation	Working outside of house	862 (24.30)	20.14	8.01	5.22	0.001
	Housewife	2679 (75.70)	18.51	7.92		
Division	Barisal	415 (11.7)	19.17	8.11	3.78	0.001
	Chittagong	674 (19.0)	18.33	7.31		
	Dhaka	626 (17.7)	17.99	8.01		
	Khulna	422 (11.9)	19.38	8.37		
	Rajshahi	436 (12.3)	18.97	8.31		
	Rangpur	446 (12.6)	20.00	8.27		
	Sylhet	522 (14.7)	19.16	7.66		
Religion	Muslim	3250 (91.8)	18.82	7.88	−1.88	0.041
	Non-Muslim	291 (8.2)	20.10	9.11		
Place of residence	Urban	1143 (32.3)	18.71	8.11	−1.00	0.061
	Rural	2398 (67.7)	19.00	7.92		
Wealth Index	Poor	1410 (39.8)	19.29	8.04	3.62	0.027
	Middle	677 (19.1)	18.98	7.95		
	Rich	1454 (41.1)	18.50	7.90		
**Anthropometric variable**						
BMI	Underweight (BMI < 18.5 kg/m^2^)	957(27.0)	18.59	7.77	1.029	0.357
	Normal weight (18.5 ≤ BMI < 25 kg/m^2^)	2021 (57.1)	19.02	7.97		
	Overweight or obese (BMI ≥ 25 kg/m^2^)	563(15.9)	19.03	8.34

**Table 2:** The Pearson’s correlation coefficients between duration of breastfeeding and selected continuous variables

Variable	Duration of breastfeeding	*p*-value
	Pearson’s correlation coefficients	
Mothers’ age	0.154	0.001
Mothers’ BMI	0.041	0.014
Total children ever born	−0.073	0.001
Mothers’ age at first birth	−0.042	0.013

### Statistical analysis

Independent sample t-test and one-way analysis of variance (ANOVA) were used to find the significant difference in DB between two and more than two groups respectively. Data was checked for the standard assumptions of independent sample t-test and ANOVA. Normality and homogeneity of cohort variances were checked using the Kolmogorov–Smirnov non-parametric test and a normal probability plot, and the Levene test respectively. Pearson’s correlation coefficient was used to find the degree of linear relationship between DB and other selected continuous variables. Finally, multiple linear regression analysis was used to identify the predictors of DB. The multiple linear regression model corresponding to each variable is: y = b_0_ + b_1_ x_1_ + b_2_x_2_ +. .. + b_k_ x_k_ + ϵ_,_ (1).

where, y is the response variable (DB), x_i_ (i = 1, 2, 3,. .., k) are the predictor variables, b_0_ is the intercept term, b_1_, b_2_,. .., b_k_ are the unknown regression coefficients, and ϵ is the error term with a N(0, σ^2^) distribution. Variation inflation factor (VIF) was used to check for the multicollinearity problem among the predictor variables in multiple linear regression analysis [24]. We used sampling weight as mentioned in BDHS-2014 for analyzing data [21]. Intra-class Correlation Coefficient (ICC) was utilized to check the variation in outcome variable DB among clusters (EAs**)** [25]. In this study, we found the value of ICC was much closed to 0 (0.0001), which meant that there was no cluster effect of DB among EAs.

We used STATA (version 11) and SPSS software (version IBM 22) for statistical analyses, and statistical significance was accepted at *p* < 0.05.

## Results

A total of 3541 mothers having children aged 6–36 months were included in the study to investigate the socio-demographic determinants of DB in Bangladesh. The mean DB among Bangladeshi mothers was 18.91 months (95% CI: 18.65–19.17) and median was 19.00 months. The Kolmogorov–Smirnov non-parametric test exhibited our dependent variable (DB) was normally distributed. In addition, the Levene test showed that the data were homogeneous.

It was found, more than 78% of mothers received ANC visits during their pregnancy period, and independent sample t-test demonstrated that the mean DB was significantly (*p* < 0.01) lower (18.63 months) among the mothers who received ANC than mothers did not receive (18.63 months). The mean DB among rural mothers (19.00 months) was somewhat longer (*p* = 0.061) than mothers living in urban environment (18.71 months). Muslim mothers provided their breast milk for a shorter duration (18.82 months) than Non-Muslim mothers (20.10 months) (*p* < 0.05). The mean DB was longer (19.10 months) among vaginal delivered mothers compared to caesarean delivered mothers (18.27 months) (*p* < 0.01). Also, mean DB (19.18 months) was longer among mothers who delivered at home than mothers delivered at hospital or clinic (18.48 months) (*p* < 0.05). Mothers working outside of house provided their breast milk to their children for significantly (*p* < 0.01) longer time (20.14 months) than housewife mothers (18.51 months). It was found that the DB was decreased with increasing the education level of mothers, and ANOVA showed that the variation of DB among mothers’ education level was significant (*p <* 0.05). Almost same pattern of DB was observed among fathers’ education level (*p* < 0.01). Highest mean value of DB was found among mothers living in Rangpur division (20.00 months) followed by Khulna (19.38 months), Barisal (19.17 months), Sylhet (19.16 months), Rajshahi (18.97 months), Chittagong (18.33 months) and Dhaka (17.99 months). The variation of DB among divisions was statistically significant (*p <* 0.01). It was found that the mean value of DB was decreased with increasing household quintile index, and the variation was significant (*p* < 0.05). The increasing tendency of mean DB was found with increasing the nutritional status of mothers, however the ANOVA demonstrated that the variation was not significant (*p* > 0.05) (Table 1).

Pearson’s correlation coefficients demonstrated that the relationship between DB and mothers’ current age (*p* < 0.01) and their BMI (*p* < 0.05) was significantly positive. The relationship between DB and total children ever born (*p <* 0.01) and mothers’ age at first born (*p <* 0.05) was significantly negative (Table 2).

The significantly difference in mean DB between two and more than two groups of categorical variables provided by t-test and ANOVA respectively, and significantly correlated continuous variables provided by Pearson’s correlation coefficients were used as the predictors in multiple linear regression model. In Table 3, we observed that VIF values of all predictors lie between 0 and 5; there was no evidence of multicollinearity problem among the predictors. The model demonstrated that the mothers’ age had significant (*p* < 0.01) positive effect on predictors (DB). However, total number of children ever born and mothers’ age at first birth had a significant (*p* < 0.01) negative effect on duration of breastfeeding. Mothers did not visit ANC who provided DB to their children averagely 1.307 months longer compared to mothers who visited ANC at least one time (*p <* 0.01). Working mothers at the outside of house provided breast milk to their children averagely 1.240 months higher than housewife mothers (*p <* 0.01). Mothers living in Barisal, Khulna, Rangpur and Sylhet divisions provided 0.972 (*p* < 0.05), 1.305 (*p <* 0.01), 1.823 (*p <* 0.01) and 1.951 (*p <* 0.01) months longer respectively than mothers living in Dhaka division. Our selected model explained the variation of dependent variable (DBF) by about 78% (Table 3).

**Table 3:** Effect of socio-demographic and anthropometric factors on duration of breastfeeding

Predictors	B	SE	*p*-value	95% CI for B		VIF
				Lower	Upper	
**Demographic and health related variable**						
Antenatal care visit, No Vs Yes^R^	1.307	0.348	0.001	0.625	1.989	1.240
Mode of delivery, Vaginal Vs Caesarian^R^	0.260	0.433	0.548	−0.589	1.110	2.025
Place of delivery, Home Vs Hospital/Clinic	0.318	0.386	0.410	−0.439	1.076	2.133
Mothers’ age at first birth	−0.637	0.061	0.001	−0.757	−0.516	2.301
Total children ever born	−1.962	0.186	0.001	−2.328	−1.597	4.226
Mothers’ age	0.660	0.047	0.001	0.568	0.752	4.355
**Socio-economic variable**						
Mothers’ educational level	0.331	0.207	0.109	−0.073	0.736	1.874
Fathers’ educational level	−0.268	0.174	0.123	−0.610	0.073	1.844
Mothers’ occupation, Working outside of house Vs Housewife^R^	1.240	0.310	0.001	0.632	1.848	1.064
Division						
Barisal Vs Dhaka^R^	0.972	0.496	0.049	−0.001	1.945	1.530
Chittagong Vs Dhaka^R^	0.647	0.433	0.136	−0.203	1.496	1.736
Khulna Vs Dhaka^R^	1.305	0.494	0.008	0.337	2.273	1.538
Rajshahi Vs Dhaka^R^	0.579	0.486	0.233	−0.374	1.533	1.533
Rangpur Vs Dhaka^R^	1.823	0.488	0.001	0.865	2.781	1.578
Sylhet Vs Dhaka^R^	1.951	0.476	0.001	1.018	2.883	1.708
Religion, Muslim Vs Non-Muslim^R^	0.707	0.477	0.139	−0.229	1.642	1.031
Wealth Index	−0.050	0.183	0.786	−0.409	0.309	1.630
**Anthropometric variable**						
Mothers’ body mass index	0.060	0.036	0.094	−0.010	0.130	1.223
***R***^**2**^**-value = 0.779**						

*N.B.* B: Regression coefficients, *SE* Standard error, *CI* Confidence interval, *VIF* Variation inflation factor, *R* Reference case

## Discussion

In this study, we found that the mean DB was 18.91 months among Bangladeshi mothers. One of the earlier studies with BDHS-1999-2000 data set reported that the mean DB among Bangladeshi mothers was 31.3 months [26]. Another study with BDHS-2004 dataset found that the mean DB of Bangladeshi mothers was 30.41 months [27]. It is observed that the mean DB is being decreased over time in Bangladesh. It may be occurred due to increase in the higher education level of women and number of caesarean delivery in Bangladesh [21]. Moreover, the average DB in Bangladesh was lower than that of other South Asian countries such as India (20.37 months) [28], Pakistan (21.8 months) [29], Sri Lanka (23.2 months) [30]. These were very old studies. One of the recent Indian studies reported that the median DB was 12 months, according to nationally representative data from the 2011–2012 Indian Human Development Survey II. They also found that the median DB had decreased by 50% from 1992 to 1993 to 2011–2012 [31]. We found that the median DB in Bangladesh was 19 months that was higher than that found in Indian study.

The mean DB was lower among the mothers who received ANC than who did not. Our results did not coincide with other studies [32] who found that DB was longer among the mothers who visited ANC. This dissimilarity was happened due to the fact that urban mothers received more ANC than rural mothers in Bangladesh [33, 34], and we found urban mothers provided their breast milk to their children averagely for a shorter period than rural mothers. Similar results were also found in India [28] that mothers residing in rural areas have longer DB compared to those living in urban areas. Most of the rural mothers delivered at home, and our study showed that the mean DB among home delivered mothers was longer than mothers who delivered at hospitals or clinics. Education, figure consciousness and availability of breast milk substitution in urban area might be the possible reason behind the shorter DB. It was also observed that the average breastfeeding period was shorter in younger mothers than older mothers. Similar results were also found in Brazil [35, 36], China [37], India [28] and Kuwait [38, 39]. This may be due to lack of experience and knowledge of younger mothers regarding breastfeeding. In addition, they might have received less counseling on benefits of breastfeeding. Our results indicated no significant difference in DB between male and female children. This finding was supported by a previous study [38]. The present study detected that average DB among educated mothers was comparatively shorter than low educated mothers. Our result coincided with other studies in Nigeria [32], Kuwait [38, 39] and India [28]. The higher educated women have more opportunities in the workforce and tend to choose their career over fertility-related matters [40]. Higher educated working mothers might not breastfeed their children for long time due to the demand of occupation [41, 42]. Educational status was one of the most important factors that influence breastfeeding practices which concords with the study conducted in Malaysia [43]. However, we found that exterior working mother breastfeed their children for long time than housewife, which was consistent with the findings of other study in Bangladesh [44]. In rural areas of Bangladesh, usually women involved in some casual works such as domestic work, jobs in cottage industries, small-scale marketing and so on. These types of working give them more time to take care of their baby and breastfeed for longer periods. Moreover, this result happened in our population due to working mothers’ education level; our data showed 11% of mothers were higher educated out of whom only 24.1% works outside at home. More research is required regarding this issue. In this study, mean DB was shorter among caesarian mothers. Similar findings were also observed in China [45] and Vietnam [46]. It is well known that mothers with C-section tend to experience longer recovery periods and more medical care [47]. Thus C-section mothers introduce solid foods for her baby and intend to stop breastfeeding earlier than mothers with normal delivery [48]. Therefore, mode of delivery can be stated as an important indicator for the DB. It was found that the mothers who delivered a large number of children had negative effect on DB in Bangladesh because their fertility returns early. In 2018, Al-Kandari also found the same results among Kuwaiti mothers [39]. The fathers’ educational level was also an important factor for DB discovered by the present study. Usually, educated male married educated female and educational level of female showed an inverse relationship with DB found in this study. This result is supported by other studies [39, 49].

It was observed that mothers who lived in Dhaka division breastfed averagely for a shorter period than other divisions in Bangladesh. Women living in Dhaka division, the Capital city of Bangladesh are comparatively more educated than women living in other divisions [50]. Our findings suggested that women who had completed at least primary education, breastfed their children for an averagely shorter period than illiterate women. Thus geographic factor can be mentioned as an important determinant for the DB.

### Strength and limitations of the study

Some studies have been done on initial and exclusive breastfeeding among Bangladeshi mothers extracting data from nationally representative dataset of BDHS-2014. Perhaps this was the first time we attempted to study on DB among Bangladeshi mothers using the latest nationally representative sample (BDHS-2014) in Bangladesh. However, there were some limitations of this study. This study was conducted using secondary data and it was bounded by the limitations of those data. Because of being a cross-sectional study, it was difficult to set up a causal relationship between socio-demographic, demographic and anthropometric factors and DB among mothers in Bangladesh. Some currently pregnant women having children aged 6–36 months could not be included in this study due to bias regarding their weight. One child (last born) aged 6–36 months was included in the study that might cause bias in estimation of duration of breastfeeding, also, mothers with children ≥6 months who were currently breastfeeding at the time of the interview were excluded from the analysis**.** From the literature review, we observed that some independent variables were very important predictor for breastfeeding but we could not include those variables such as ethnicity, birth order, gestational age etc. [51]. Though we used the latest nationally representative data six years have already been passed. Clearly, more research is required with duration of breastfeeding among Bangladeshi mothers using new nationally representative data.

## Conclusions

In the present study, we tried to determine the factors which were related to the duration of breastfeeding among mothers in Bangladesh using nationally representative data collected by BDHS-2014. Our selected statistical technique/models provided that ANC, religion, mode of delivery, parents’ education, geographic location (division), mothers’ age, mothers’ BMI, total ever born children, mothers’ age at first birth and household wealth quintile were associated factors of duration of breastfeeding among Bangladeshi mothers. The socio-demographic factors related to overall duration of breastfeeding can be a valuable appliance when planning local actions and policies aimed at improving breastfeeding duration. The present study indicated that the breastfeeding-promotion programme such as a regular maternal, newborn, child and adolescent health (MNCAH) program, world breastfeeding week, national nutrition program (NNP) of Ministry of Health and Family Welfare (MOHFW) in Bangladesh should address our findings. Government should take proper care and more attention about the maternal health benefit of breastfeeding and encourage mothers to breastfeed their child for at least 6 months. Improving mothers’ knowledge and understanding of the breastfeeding was also recommended.

## Data Availability

The BDHS-2014 datasets and relevant materials are freely available at https://dhsprogram.com/data/dataset/Bangladesh_Standard-DHS_2014.cfm?flag=0.

## Abbreviations

ANC: Antenatal Care
ANOVA: Analysis of variance
BBS: Bangladesh Bureau of Statistics
DB: Duration of breastfeeding
BDHS: Bangladesh Demographic and Health Survey
BMI: Body Mass Index
CI: Confidence Interval
EA: Enumeration area
NIPORT: National Institute of Population Research and Training
PSU: Primary Sampling Uunit
SDGs: *Sustainable Development Goals*
SE: Standard error
SPSS: Statistical Package For Social Sciences
UNICEF: United Nations International Children’s Emergency Fund
VIF: Variance inflation factor
WHO: World Health Organization
WI: Wealth Index
